# Clinicopathological features and prognostic significance of *CTNNB1* mutation in low-grade, early-stage endometrial endometrioid carcinoma

**DOI:** 10.1007/s00428-021-03176-5

**Published:** 2021-08-21

**Authors:** Ignacio Ruz-Caracuel, Álvaro López-Janeiro, Victoria Heredia-Soto, Jorge L. Ramón-Patino, Laura Yébenes, Alberto Berjón, Alicia Hernández, Alejandro Gallego, Patricia Ruiz, Andrés Redondo, Alberto Peláez-García, Marta Mendiola, David Hardisson

**Affiliations:** 1 Department of Pathology, Hospital Universitario La Paz, IdiPAZ, 28046 Madrid, Spain; 2 Present Address: Department of Pathology, Hospital Universitario Ramón Y Cajal, IRYCIS, 28034 Madrid, Spain; 3 Translational Oncology Research Laboratory, Hospital La Paz Institute for Health Research (IdiPAZ), 28046 Madrid, Spain; 4 Center for Biomedical Research in the Cancer Network (Centro de Investigación Biomédica en Red de Cáncer, CIBERONC), Instituto de Salud Carlos III, 28046 Madrid, Spain; 5 Department of Medical Oncology, Hospital Universitario La Paz, IdiPAZ, 28046 Madrid, Spain; 6 Present Address: Department of Medical Oncology, Hospital Universitario Rey Juan Carlos, Móstoles, 28933 Madrid, Spain; 7 Molecular Pathology and Therapeutic Targets Group, Hospital La Paz Institute for Health Research (IdiPAZ), Paseo de la Castellana, 261, 28046 Madrid, Spain; 8 Department of Obstetrics & Gynecology, Hospital Universitario La Paz, IdiPAZ, 28046 Madrid, Spain; 9 Faculty of Medicine, Universidad Autónoma de Madrid, 28029 Madrid, Spain

**Keywords:** Endometrial cancer, Endometrioid carcinoma, Low grade, Prognosis, *CTNNB1* mutation, Beta-catenin, LEF1, Microsatellite instability

## Abstract

Low-grade and early-stage endometrioid endometrial carcinomas (EECs) have an overall good prognosis but biomarkers identifying patients at risk of relapse are still lacking. Recently, *CTNNB1* exon 3 mutation has been identified as a potential risk factor of recurrence in these patients. We evaluate the prognostic value of *CTNNB1* mutation in a single-centre cohort of 218 low-grade, early-stage EECs, and the correlation with beta-catenin and LEF1 immunohistochemistry as candidate surrogate markers. *CTNNB1* exon 3 hotspot mutations were evaluated by Sanger sequencing. Immunohistochemical staining of mismatch repair proteins (MLH1, PMS2, MSH2, and MSH6), p53, beta-catenin, and LEF1 was performed in representative tissue microarrays. Tumours were also reviewed for mucinous and squamous differentiation, and MELF pattern. Nineteen (8.7%) tumours harboured a mutation in *CTNNB1* exon 3. Nuclear beta-catenin and LEF1 were significantly associated with *CTNNB1* mutation, showing nuclear beta-catenin a better specificity and positive predictive value for *CTNNB1* mutation. Tumours with *CTNNB1* exon 3 mutation were associated with reduced disease-free survival (*p* = 0.010), but no impact on overall survival was found (*p* = 0.807). The risk of relapse in tumours with *CTNNB1* exon 3 mutation was independent of FIGO stage, tumour grade, mismatch repair protein expression, or the presence of lymphovascular space invasion. *CTNNB1* exon 3 mutation has a negative impact on disease-free survival in low-grade, early-stage EECs. Nuclear beta-catenin shows a higher positive predictive value than LEF1 for *CTNNB1* exon 3 mutation in these tumours.

**Graphical abstract:**

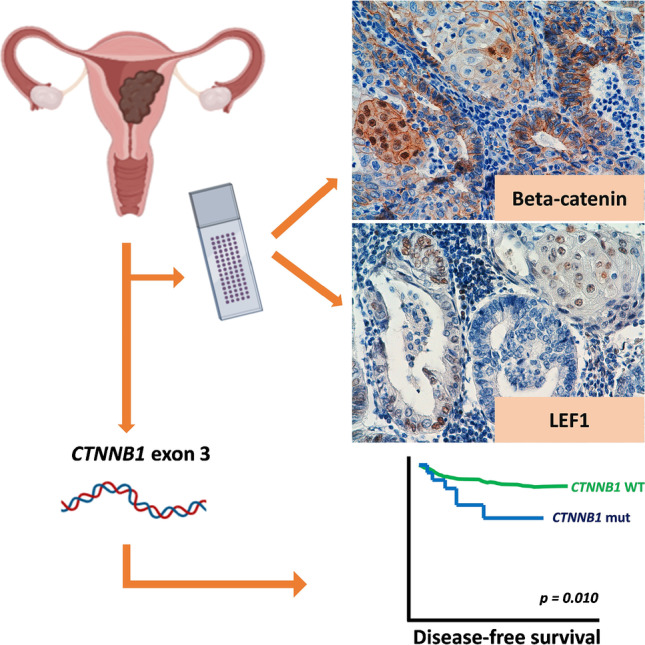

**Supplementary Information:**

The online version contains supplementary material available at 10.1007/s00428-021-03176-5.

## Introduction

Endometrial cancer is the leading cause of gynaecological cancer and the third most frequent cancer in women [1]. Low-grade (histologic grade 1 or 2), early-stage (FIGO stage I–II) endometrioid endometrial carcinomas (EECs) have an overall good prognosis and are classified into low- or intermediate-risk categories using clinicopathological features [2–4]. However, between 4 and 13% of these patients develop local or distance relapses [5, 6]. Early identification of these cases remains challenging.

Using data from endometrioid carcinomas of the TCGA project, Liu et al. identified *CTNNB1* exon 3 mutations as a potential risk factor of recurrence in low-grade, early-stage EECs [7]. This was later confirmed in most but not all studies [8–12]. Most of these reports are case–control studies [8, 11, 12] and there is a lack of consecutive single-centre series with extensive morphological evaluation. Apart from the presence of squamous morules [13], little is known about other morphological features associated with the presence of this mutation.

Tumours carrying *CTNNB1* exon 3 mutations activate the Wnt/beta-catenin through aberrant translocation of beta-catenin from the membrane to the nucleus, where it can be identified using immunohistochemistry (IHC) [14]. Beta-catenin IHC has been repeatedly evaluated as a candidate surrogate marker of *CTNNB1* mutation with variable sensitivity and specificity [15]. Another candidate that has not yet been evaluated is LEF1, a nuclear effector of the Wnt/beta-catenin pathway [16], overexpressed in *CTNNB1*-mutated tumours according to the Clinical Proteomic Tumour Analysis Consortium (CPTAC) proteogenomic characterisation of endometrial cancer [17].

The main objective of this study was to evaluate the prognostic impact of *CTNNB1* exon 3 hotspot mutation in a large series of low-grade, early-stage EECs. Additionally, we aimed to analyse the potential correlation of LEF1 and beta-catenin IHC with *CTNNB1* exon 3 hotspot mutation, and to identify morphological parameters that could be predictive of *CTNNB1* mutation in these tumours.

## Materials and methods

### Cohort selection

A single-centre retrospective cohort of EECs was identified from patients fulfilling the following criteria: primary EEC grade 1 or 2, FIGO 2009 stage I or II, hysterectomy and bilateral salpingo-oophorectomy performed between January 2003 and December 2015 at Hospital Universitario La Paz (Madrid, Spain), oncological follow-up at the same centre, and available tissue for exon 3 *CTNNB1* mutation analysis. Clinicopathological features were retrieved from the pathological reports and the clinical records of the patients.

The study was approved by the local Ethics Committee (code HULP: PI-3108) and was conducted in accordance with ethical standards of the Helsinki Declaration of the World Medical Association.

### Histopathological analysis and immunohistochemistry

All tumour slides were reviewed by one author (I.R.-C.) (mean ± standard deviation: 6.84 ± 3.23, range: 1–24) and discrepancies with original reports were solved with an experienced gynaecopathologist (D.H.). In addition, the following morphological variables were evaluated: mucinous differentiation; squamous differentiation; and the presence of microcystic, elongated, and fragmented (MELF) pattern of myoinvasion. Mucinous differentiation is defined as the presence of any percentage of cells with intracytoplasmastic mucin. Squamous differentiation is defined as any kind of squamous metaplasia, including morular metaplasia. MELF is defined as the presence of slit-like, microcystic, and/or individual tumour cells with eosinophilic change that are admixed with inflammation at the leading edge of the tumour [18]. Moreover, it was annotated if MELF pattern appeared as a predominant pattern or as a secondary pattern, as previously described [19].

Two representative central areas from each tumour were marked on haematoxylin–eosin slides and tissue microarrays (TMAs) containing cores of 1.2 mm were constructed using a TMA workstation (Beecher Instruments, Silver Spring, MD, USA), as described previously [19]. IHC was performed on 4-µm sections of the TMA blocks by the Envision method (Dako-Agilent, Glostrup, Denmark) in an automated Omnis platform (Dako-Agilent) according to the manufacturer’s instructions with the following monoclonal antibodies: beta-catenin (β-catenin-1, Dako-Agilent; prediluted), LEF1 (EP310, Cell Marque, Sigma-Aldrich, Darmstadt, Germany; 1:100), and p53 (clone DO-7, Dako-Agilent; prediluted). DNA mismatch repair (MMR) proficiency was determined using the following primary antibodies: MLH1 (clone ES05, Dako-Agilent; prediluted), PMS2 (clone EP51, Dako-Agilent; prediluted), MSH2 (clone FE11, Dako-Agilent; prediluted), and MSH6 (clone EP49, Dako-Agilent; prediluted). Beta-catenin was evaluated as positive when any percentage of nuclear staining in tumour cells in any of the two cores was present and negative when no nuclear staining was observed. LEF1 evaluation on TMA was performed using the Allred score [20]. Briefly, the Allred score uses a visual scale to measure the percentage of nuclear staining from 0 to 5 and the intensity of nuclear staining from 0 to 3. The final score is the sum of both variables. Final Allred score in tumours with two cores analysed was the mean of both values. An Allred score of ≥ 3 was considered positive (LEF1 overexpression), to exclude cases with focal LEF1 expression sampled from the myoinvasive front. DNA MMR proteins (MLH1, PMS2, MSH2, and MSH6) were evaluated as positive (MMR-proficient) when any nuclear staining was present, irrespective of staining intensity, and negative (MMR-deficient) when no nuclear immunostaining was found. Cases were considered MMR-deficient when at least one MMR protein was negative. p53 immunostaining was interpreted according to current recommendations: tumours showing variable nuclear expression and intensity were noted as wild-type, tumours showing strong nuclei positivity in more than 80% of tumour cells or showing cytoplasmic staining in more than 80% of tumour cells were noted as aberrant-mutation pattern, and tumours showing complete absence of nuclei positivity were noted as null-mutation pattern [21]. In doubtful cases for MMR proteins and/or p53 evaluation, whole slide IHC was performed. Whole slide beta-catenin was also studied in tumours harbouring *CTNNB1* mutation with absent beta-catenin staining on TMA. In addition to TMA, whole slide LEF1 expression was also evaluated in 21 non-selected cases of our series of EC.

### Mutation testing of CTNNB1

Selected formalin-fixed paraffin-embedded (FFPE) blocks containing > 50% of viable tumour tissue were used to extract DNA by QIAamp FFPE tissue kit (Qiagen) and used for PCR and Sanger sequencing. A 226 bp fragment of *CTNNB1* exon 3, encompassing the region of GSK-3β phosphorylation site, was amplified with these specific primers (5′-3′): GATTTGATGGAGTTGGACATGG and TGTTCTTGAGTGGAAGGACTGAG.

### Follow-up and statistical analysis

Tumour relapse was defined as the occurrence of local tumour recurrence, lymph node metastasis, and/or distant metastasis. Disease-free survival (DFS) was defined as the time from the date of diagnosis to relapse or death due to any cause. Overall survival (OS) was defined as the time from the date of diagnosis to death due to any cause.

Quantitative results were expressed as mean ± standard deviation. The chi-squared or Fisher’s exact test was used to evaluate the association between qualitative variables. Mann–Whitney’s *U* test was used to evaluate the association between quantitative variables in both groups. Prognostic clinicopathological factors in *CTNNB1* exon 3 mutated and non-mutated tumours in recurrence and non-recurrence groups were statistically analysed with univariate logistic regression. According to these results, multivariate logistic regression was modelled using the significant parameters (*p* < 0.05) from the univariate analysis and the age ≥ 60 years. DFS and OS data were plotted in Kaplan–Meier curves, and the log-rank test was used to compare these parameters.

Data were analysed using the statistical software IBM SPSS v19 (Chicago, IL, USA). Differences were considered significant with *p* values < 0.05.

## Results

### Cohort characteristics

A total of 218 low-grade, early-stage EECs fulfilled the inclusion criteria. Table 1 shows the clinicopathological characteristics of the tumours grouped by *CTNNB1* exon 3 mutational status. Nineteen (8.7%) tumours harboured a mutation in *CTNNB1* exon 3 (Supplementary table 1).

**Table 1:** Clinicopathological characteristics of the patients

		***CTNNB1* exon 3 wild-type**	***CTNNB1* exon 3 mutated**	***p*-value**
Total number	218	199 (91.3%)	19 (8.7%)	
**Age (yr)**	64.04 ± 10.38	64.19 ± 10.06	62.47 ± 13.54	0.251
**FIGO stage**				
IA	149 (68.3%)	139 (69.8%)	10 (52.6%)	0.304
IB	61 (28.0%)	53 (26.6%)	8 (42.1%)	
II	8 (3.7%)	7 (3.5%)	1 (5.3%)	
**Adjuvant treatment**				
None	153 (70.2%)	141 (70.9%)	12 (63.2%)	0.484
Radiotherapy	65 (29.8%)	58 (29.1%)	7 (36.8%)	
**Tumour grade**				
G1	165 (75.7%)	149 (74.9%)	16 (84.2%)	0.575
G2	53 (24.3%)	50 (25.1%)	3 (15.8%)	
**Lymphovascular space invasion**				
Present	38 (17.4%)	32 (16.1%)	6 (31.6%)	0.110
Absent	180 (82.6%)	167 (83.9%)	13 (68.4%)	
**Tumour relapse**				
Yes	27 (12.4%)	21 (10.6%)	6 (31.6%)	**0.018**
No	191 (87.6%)	178 (89.4%)	13 (68.4%)	
**Squamous differentiation**				
Present	81 (37.2%)	68 (34.2%)	13 (68.4%)	**0.003**
Absent	137 (62.8%)	131 (65.8%)	6 (31.6%)	
**Mucinous differentiation**				
Present	58 (26.6%)	57 (28.6%)	1 (5.3%)	**0.028**
Absent	160 (73.4%)	142 (71.4%)	18 (94.7%)	
**MELF pattern**				
Present	29 (13.3%)	28 (14.1%)	1 (5.3%)	0.480
Absent	189 (86.7%)	171 (85.9%)	18 (94.7%)	
**DNA MMR protein expression**				
Proficient	163 (75.5%)	144 (73.1%)	19 (100%)	Not calculated
Deficient	53 (24.5%)	53 (26.9%)	0	
**p53-mutant pattern**				
Present	8 (3.7%)	8 (4.1%)	0	Not calculated
Absent	206 (96.3%)	187 (95.9%)	19 (100%)	
**Nuclear beta-catenin**				
Present	24 (11.3%)	13 (6.7%)	11 (57.9%)	** < 0.001**
Absent	188 (88.7%)	180 (93.3%)	8 (42.1%)	
LEF1 overexpression*				
Present	75 (36.2%)	62 (32.8%)	13 (72.2%)	**0.001**
Absent	132 (63.8%)	127 (67.2%)	5 (27.8%)	

Bold values indicate statistically significant *p* values (*p* < 0.05)

*Allred score ≥ 3; *MMR*, mismatch repair protein; *MELF*, microcystic, elongated, and fragmented

There were no significant differences between *CTNNB1* exon 3 mutant and wild-type cases regarding established risk parameters, such as age, FIGO stage, tumour grade, and the presence of lymphovascular space invasion (LVSI) (Table 1). Moreover, there were no differences regarding adjuvant radiotherapy (*p* = 0.484). No patient received adjuvant chemotherapy. Interestingly, *CTNNB1* exon 3 mutations were associated with higher risk of tumour relapse. Thus, there were 6 patients (31.6%) who developed tumour relapse in the *CTNNB1*-mutated group compared with 21 patients (10.6%) in the *CTNNB1* wild-type group (*p* = 0.018). Relapses in tumours with *CTNNB1* exon 3 mutation were locoregional in 4 patients and distant in 2 patients (metastases in mediastinal lymph nodes in one case, and multiple metastases in bone and liver in the other case).

*CTNNB1* exon 3 mutations were significantly associated with certain morphological features, such as the presence of squamous differentiation (*p* = 0.003) and the absence of mucinous differentiation (*p* = 0.028). Only one tumour with *CTNNB1* exon 3 mutation showed a MELF pattern of myoinvasion.

A total of 216 (99%) and 214 (98%) cases were evaluable for DNA MMR protein expression and p53 by IHC, respectively. Failed cases were considered when any of the IHC for DNA MMR proteins showed no reactivity in the TMA and in the whole section. Eight tumours (3.9%) showed a p53-mutant pattern (five cases showed diffuse overexpression of the p53 protein, and the remaining three tumours showed a null pattern confirmed by whole slide IHC). No case showed an abnormal cytoplasmic p53 pattern. In our series, all tumours with *CTNNB1* exon 3 mutation were DNA MMR-proficient. We found that *CTNNB1* exon 3 mutation was mutually exclusive with alterations in p53 and DNA MMR.

### Prognostic significance of CTNNB1 exon 3 mutation in low-grade, early-stage EECs

**Fig. 1:**
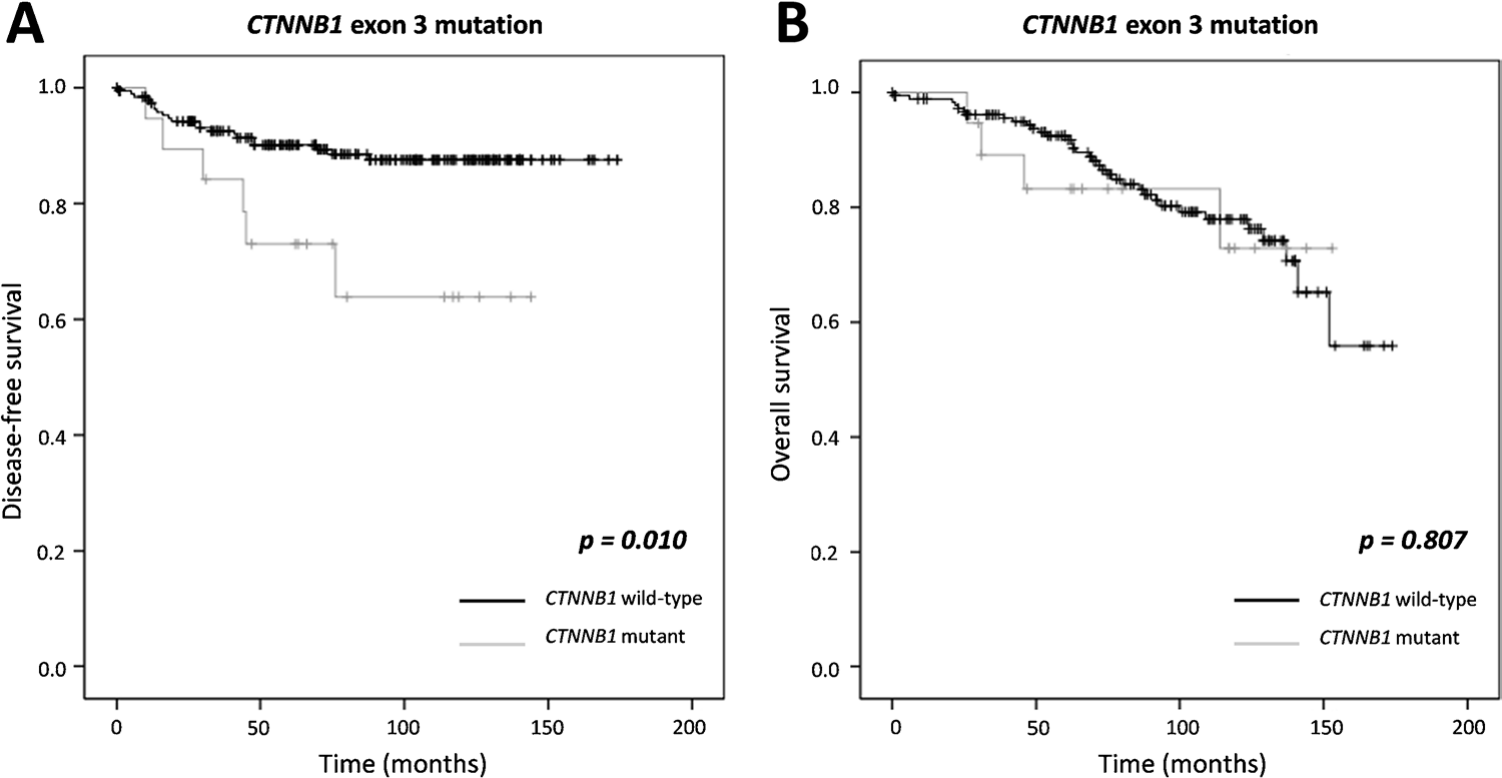
Kaplan–Meier curves for disease-free survival (DFS) and overall survival (OS) in low-grade, early-stage EECs. DFS (**A**) and OS (**B**) according to the presence of *CTNNB1* exon 3 mutation

In our series, median follow-up was 80.50 months. The mean DFS was 80.37 ± 44.05 (range, 0–174) months and mean OS was 84.04 ± 41.68 (range, 1–174) months. Patients with *CTNNB1* exon 3 mutation had a mean DFS of 73.79 ± 41.59 (range, 10–144) months compared with 81.00 ± 44.32 (range, 0–174) months for patients with wild-type *CTNNB1* exon 3 tumours (*p* = 0.010) (Fig. 1A). In contrast, OS was not significantly different for patients with or without *CTNNB1* exon 3 mutation (83.37 ± 43.00 (range, 26–153) months vs 84.10 ± 41.66 (range, 1–174) months, respectively, p = 0.807) (Fig. 1B).

In the univariate logistic regression model, tumours harbouring a *CTNNB1* exon 3 mutation had a relative risk of relapse of 3.912 (*p* = 0.012) (Table 2). Classical clinicopathological parameters, such as tumour grade 2 vs grade 1 (*p* = 0.038), FIGO stage ≥ IB (*p* < 0.001), and the presence of LVSI (*p* = 0.001), were associated with tumour relapse in the univariate logistic regression model (Table 2).

**Table 2:** Univariate and multivariate analyses of odds ratios in the logistic regression model with relapse as the dependent variable

Parameter	Odds ratio (CI 95%)	*p*-value
**Univariate logistic regression model**		
Age ≥ 60 years	2.662 (0.966–7.340)	0.058
Tumour grade 2	2.439 (1.052–5.653)	**0.038**
FIGO stage ≥ IB	3.347 (1.744–6.421)	** < 0.001**
LVSI	4.176 (1.751–9.957)	**0.001**
DNA MMR-deficient protein expression	2.406 (1.038–5.578)	**0.041**
*CTNNB1* exon 3 mutation	3.912 (1.345–11.380)	**0.012**
**Multivariate logistic regression model**		
Age ≥ 60 years	2.298 (0.734–7.191)	0.153
Tumour grade 2	1.732 (0.666–4.500)	0.260
FIGO stage ≥ IB	3.129 (1.197–8.178)	**0.020**
LVSI	2.166 (0.786–5.968)	0.135
DNA MMR-deficient protein expression	2.361 (0.868–6.421)	0.092
*CTNNB1* exon 3 mutation	5.000 (1.334–18.745)	**0.017**

Bold values indicate statistically significant *p* values (*p* < 0.05)

*LVSI*, lymphovascular space invasion; *MMR*, mismatch repair

A multivariate logistic regression model was calculated including the parameters reaching statistical significance (*p* < 0.05) in the univariate analysis; age ≥ 60 years was added to the multivariate model because it is a known parameter associated with relapse in endometrial cancer [2]. In the multivariate analysis, only *CTNNB1* exon 3 mutation and FIGO stage ≥ IB appeared to be independently and significantly associated with tumour recurrence (*p* = 0.017 and *p* = 0.020, respectively) (Table 2).

### Pathological features associated with CTNNB1 mutation and correlation with beta-catenin and LEF1 immunohistochemistry

**Fig. 2:**
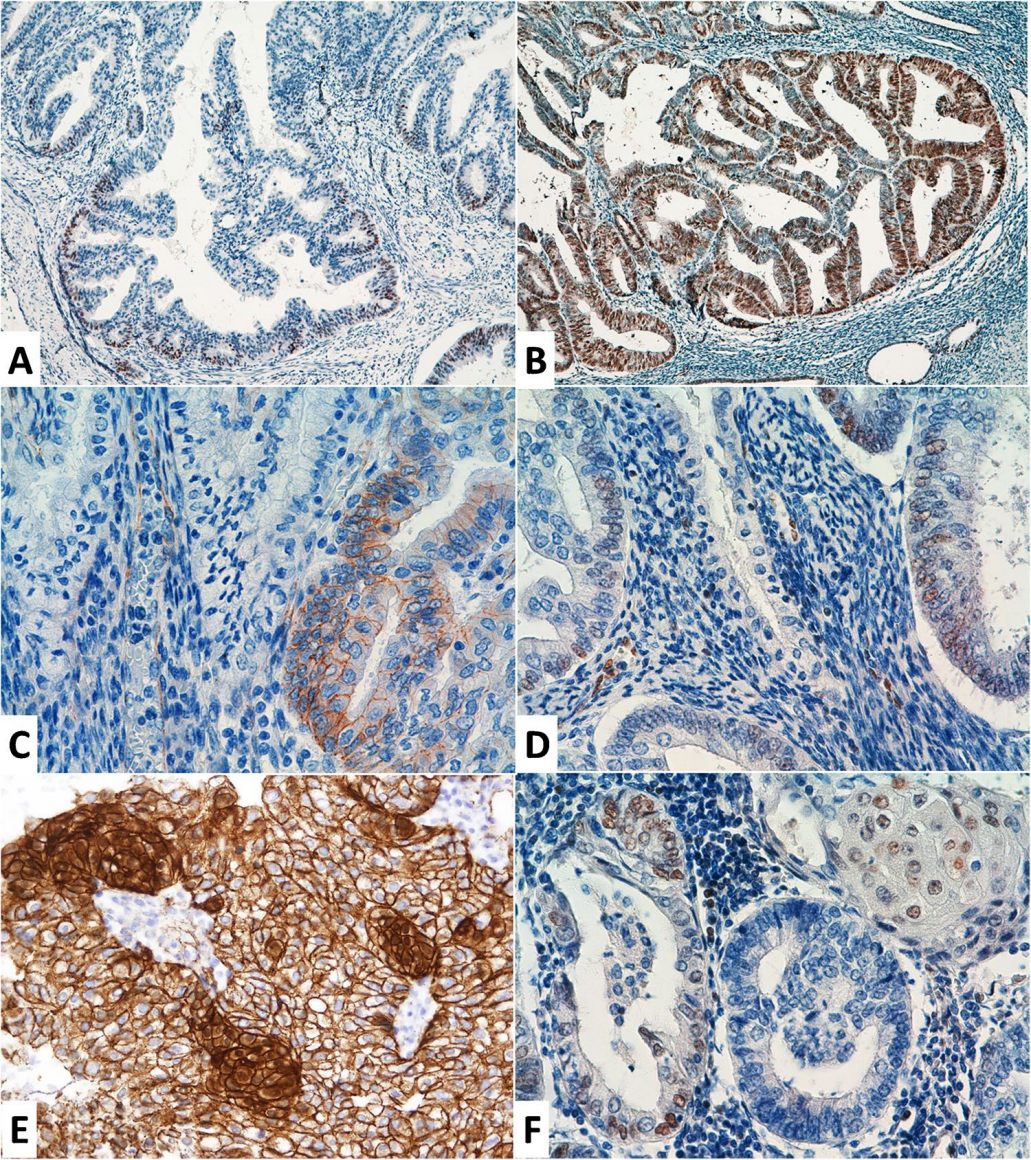
Immunohistochemical expression of LEF1 and beta-catenin. Representative areas from whole slide images of LEF1 staining showing a myoinvasive front restricted staining (**A**), in contrast to an overexpression pattern (**B**). Paired examples of a tumour showing a membranous beta-catenin staining pattern without nuclear expression (**C**) and positive LEF1 nuclear staining (**D**). Paired examples of a tumour showing positive nuclear beta-catenin (**E**) and LEF1 expression (**F**)

LEF1 immunostaining was evaluable in 207 out of 218 cases; the remaining 11 cases were not evaluable due to technical issues. LEF1 nuclear expression was observed in 92 (44.4%) out of 207 cases and an Allred score of ≥ 3 was observed in 75 (36.2%) tumours. Allred score was significantly higher in tumours with *CTNNB1* mutation (3.36 ± 2.26) compared with *CTNNB1* wild-type tumours (1.74 ± 2.18) (*p* = 0.004). Additionally, LEF1 IHC was performed in representative whole slides of 21 tumours. Two different patterns of LEF1 staining were identified. Firstly, eleven tumours showed LEF1 positive staining restricted to the invasive front (Fig. 2A). In contrast, ten tumours showed a diffuse or patchy overexpression of LEF1 in addition to that present at the invasive front (Fig. 2B).

In our TMA series, twenty-four (11.3%) out of 212 evaluated tumours showed nuclear expression of beta-catenin. Six cases could not be evaluated due to technical issues. There was a positive significant association between the nuclear expression of beta-catenin and the overexpression of LEF1 (Allred score ≥ 3) (*p* = 0.001). However, LEF1 and beta-catenin were not always expressed in the same areas. Representative images of nuclear beta-catenin and LEF1 protein expression are shown in Fig. 2C–F.

To identify pathological parameters that can aid to identify *CTNNB1*-mutated tumours, sensitivity and specificity of pathological features to predict *CTNNB1* mutation were calculated in our series (Table 3). The most sensitive parameters were the absence of mucinous differentiation and the absence of MELF pattern. In contrast, the most specific parameter was nuclear beta-catenin IHC, followed by the presence of LVSI (Table 3).

**Table 3:** Sensitivity and specificity of pathological parameters to predict *CTNNB1* exon 3 mutation

	*N*	Sensitivity	Specificity
Tumour grade 2	218	0.16	0.75
LVSI	218	0.32	0.84
Squamous differentiation	218	0.68	0.66
Absence of mucinous differentiation	218	0.95	0.29
Absence of MELF pattern	218	0.95	0.14
Nuclear beta-catenin IHC	212	0.58	0.93
LEF 1 overexpression*	207	0.67	0.72

*Allred score ≥ 3; *LVSI*, lymphovascular space invasion; *PPV*, positive predictive value; *NPV*, negative predictive value; *MELF*, microcystic, elongated, and fragmented

Eleven (57.9%) and 13 (72.2%) cases with *CTNNB1* exon 3 mutation showed nuclear expression of beta-catenin and overexpression of LEF1, respectively. Overexpression of LEF1 showed a higher sensitivity to predict *CTNNB1* exon 3 mutation compared to nuclear beta-catenin (Table 3). In contrast, nuclear beta-catenin showed a higher specificity (Table 3). The eight cases with *CTNNB1* exon 3 mutation and absent nuclear beta-catenin expression in TMA were studied in representative whole slides, showing focal nuclear beta-catenin in four of them (50%).

DNA MMR protein expression and p53 pattern predictive values for *CTNNB1* mutation were not calculated, as there were no cases harbouring these alterations (Table 1).

## Discussion

Our results demonstrate that *CTNNB1* exon 3 mutation is significantly associated with decreased DFS in patients with low-grade, early-stage EECs. This association is independent of other prognostic parameters currently used to risk stratification of patients such as age, tumour grade, FIGO stage, and LVSI in this population [3]. In contrast, no effect in OS was observed.

The association of *CTNNB1* exon 3 mutation with recurrence in early-stage endometrial carcinomas was firstly described by Liu et al. [7]. These results were later confirmed by most authors [8–11], However, an important limitation of most of these studies is that they were designed as case–control studies [8, 10–12, 22], which may not be representative of the full spectrum of a complete series. In this sense, our study is one of the largest including a consecutive single-centre population of low-grade, early-stage EECs. Surprisingly, the percentage of *CTNNB1*-mutated tumours is lower (8.7%) than that described in previous series [7–10]; however, we could not find any reason that could explain these discrepancies.

Interestingly, all tumours with *CTNNB1* exon 3 hotspot mutations in our series were DNA MMR-proficient. These results partly agree with those of Moroney et al. [23] showing a higher number of cases with *CTNNB1* exon 3 mutation in microsatellite stable tumours in a case–control descriptive study including grade 1, early-stage EECs. Moreover, in the TCGA study, 53% of tumours in the copy-number low and 19% in the microsatellite instability (hypermutated) categories harboured mutations in the *CTNNB1* gene [24]. This suggests independent tumorigenic pathways, as already described in the literature [25]. *CTNNB1* exon 3 mutation is mutually exclusive with mutant pattern p53 expression assessed by IHC, in accordance with data from the TCGA cohort that demonstrated few *CTNNB1* mutations in the copy-number high subgroup, which is defined molecularly by *TP53* mutation [24]. In this regard, a fifth molecular subgroup has been proposed comprising EECs with *CTNNB1* mutations that may have an intermediate prognosis [26]. Moreover, *CTNNB1* mutations are considered in PORTEC4a, a clinical trial designed to assess histomolecular classification to assign different treatments in stage I and II EECs [27]. In addition, *CTNNB1* mutation may be useful as a predictive biomarker. In this sense, it has been recently shown that spindle assembly checkpoint kinase TTK inhibitors, which are currently in phase I clinical trials, are more effective in *CTNNB1* mutant cell lines than in *CTNNB1* wild-type lines [28].

Tumours with both nuclear beta-catenin and nuclear LEF1 protein expression were significantly associated with *CTNNB1* mutation. We assume that the low sensitivity of nuclear beta-catenin expression in detecting *CTNNB1* mutation may be mainly due to the methodology used in the study. Thus, TMA may miss some areas of the tumour with focal nuclear expression of beta-catenin. In our series, half of the tumours harbouring *CTNNB1* mutation and absent TMA beta-catenin expression showed focal nuclear expression in the whole slide. In this sense, Kim et al. recently reported that nearly half of endometrial carcinomas with nuclear expression of beta-catenin showed this pattern in only 5–10% of tumour cells [14].

LEF1 is a transcription factor, whose ability to transactivate is dependent on the arrangement and occupancy of the protein-binding sites surrounding the LEF1 binding site [29]. Beta-catenin is one of the main proteins that interacts with LEF1 causing its transactivation [29]. When LEF1 binds to beta-catenin, it activates epithelial-to-mesenchymal transition and cell proliferation [17]. Hence, LEF1 has been implicated in tumorigenesis and progression of several neoplasms [17], including endometrial cancer, where it has been suggested that it may have a potential value as a prognostic biomarker [30]. LEF1 IHC has been recently introduced as a useful tool in the diagnosis of solid-pseudopapillary neoplasm of the pancreas, a tumour with a gain-of-function mutation in *CTNNB1* [31]. As expected, tumours harbouring *CTNNB1* exon 3 mutation showed a higher LEF1 Allred score in accordance with proteomic data from CPTAC study [17]. However, to the best of our knowledge, there are no studies that analyse the correlation between the expression of LEF1 and beta-catenin. According to our data, LEF1 correlates with *CTNNB1* exon 3 mutation with a slightly higher sensitivity than nuclear beta-catenin expression although the latter shows higher specificity. Considering these results, LEF1 IHC does not provide additional benefits to beta-catenin IHC.

Another novel finding is our observation of the negative association between *CTNNB1* mutation and the presence of MELF pattern in these tumours. MELF pattern of myoinvasion was less common in *CTNNB1* mutant than wild-type tumours (5.3% vs 14.1%), and this was a focal finding in the only case with *CTNNB1* mutation. This suggests that MELF pattern of myoinvasion could reflect a molecular pathway independent of *CTNNB1* mutation. In this regard, it is interesting that the MELF pattern of myoinvasion has been associated with the presence of single-cell metastases in locoregional lymph nodes without an impact on prognosis [32, 33]. In contrast, tumours with *CTNNB1* exon 3 mutation are prone to develop tumour recurrence. An additional finding of our study is the negative association between *CTNNB1* mutation and the presence of mucinous differentiation. Both features could be useful to select those ECs to test for *CTNNB1* mutation based on pathological assessment.

Squamous differentiation (including morular alteration) showed a positive significant association with *CTNBB1* exon 3 hotspot mutations. This is to be expected because morular alteration, the most frequent squamous metaplasia in EECs, shows strong and diffuse nuclear beta-catenin expression associated with *CTNNB1* mutations in endometrial carcinomas [13, 34] and its precursors [32, 35].

One of the limitations of this study is the relatively low number of recurrences, a common problem in studies of low-grade, early-stage EECs due to the low incidence of adverse events in these tumours. We must also take into account that for both MMR protein expression and p53 immunohistochemistry subclonal abnormal expression exists in a considerable number of cases; it is possible that this specific pattern may be missed when using a TMA approach, as we did in our study. Moreover, beta-catenin immunohistochemistry is known to show extensive intratumoral heterogeneity, and this may limit the interpretation of this protein in TMA.

In summary, we found that *CTNNB1* exon 3 mutation is associated with decreased DFS in low-grade, early-stage EECs. Moreover, the risk of relapse in tumours with *CTNNB1* exon 3 mutation is independent of other clinicopathological prognostic factors, such as FIGO stage, tumour grade, DNA MMR-deficient protein expression, or presence of LVSI. This fact reinforces that *CTNNB1*-mutated tumours may be considered a fifth group of intermediate prognoses among low-grade, early-stage EECs, apart from already established TCGA molecular groups [24]. Morphological parameters such as the absence of mucinous differentiation and the absence of MELF pattern showed high sensitivity to identify *CTNNB1*-mutated tumours. IHC showing nuclear beta-catenin and LEF1 overexpression are associated with *CTNNB1* exon 3 mutation in these tumours, having nuclear beta-catenin a better specificity for *CTNNB1* mutation.

## Supplementary Information

Below is the link to the electronic supplementary material.

Supplementary file1 (DOCX 23 KB)

## Data Availability

Data is available upon author request.
