# Supplementary material for: Clinicopathological features and prognostic significance of CTNNB1 mutation in low-grade, early-stage endometrial endometrioid carcinoma
Source: Virchows Arch. 2021 Aug 21;479(6):1167–76. doi: 10.1007/s00428-021-03176-5 (PMC8724178; doi:10.1007/s00428-021-03176-5)
Supplement: Supplementary file 1 — Supplementary file1 (DOCX 23 KB) [file 428_2021_3176_MOESM1_ESM.docx]

**Supplementary Table 1.** Identified *CTNNB1* exon 3 mutations.

| **CTNNB1 mutation** | **Number of tumours** |
| --- | --- |
| c.94G>T; p.D32Y; COSM5661 | 2 |
| c.98C>T; S33F; COSM5669 | 3 |
| c.101G>T; p.G34V; COSM5670 | 5 |
| c.110C>T; p.S37F; COSM5662 | 3 |
| c.121A>G; p.T41A; COSM5664 | 3 |
| c.122C>T; p.T41I; COSM5676 | 2 |
| c.133T>C; p.S45P; COSM5663 | 1 |
